# Psychiatric Comorbidities in Parkinson’s Disease: A Moroccan Perspective on Anxiety and Depression

**DOI:** 10.3390/diseases13110361

**Published:** 2025-11-06

**Authors:** Khaoula Elcadi, Oussama Cherkaoui Rhazouani, Nissrine Louhab, Najib Kissani, Mohamed Chraa

**Affiliations:** 1 Clinical Experimental and Environmental Neuroscience Laboratory, Faculty of Medicine and Pharmacy of Marrakech, Cadi Ayaad University, Marrakech 40000, Morocco; 2 Neurology Department, Military Hospital Avicenne, Marrakech 40000, Morocco; cheroussama28@gmail.com; 3 Neuroscience Research Laboratory, Marrakech Medical School, Cadi Ayyad University, Marrakech 40000, Morocco; 4 Department of Neurology, Mohammed VI University Medical Center, Marrakesh 40000, Morocco; nislouhab@gmail.com (N.L.); najibkis@gmail.com (N.K.)

**Keywords:** anxiety, Parkinson’s disease, depression, quality of life

## Abstract

**Background/Objectives:** An individual’s quality of life is greatly impacted by the motor and non-motor symptoms of Parkinson’s disease (PD), which include anxiety and depression. Using the Hospital Anxiety and Depression Scale (HADS), this study sought to determine the prevalence of anxiety and depression in Moroccan patients with Parkinson’s disease (PD) and investigate any possible associations with clinical characteristics and pharmacological treatment. **Methods:** The HADS was used to assess 100 PD patients in total. Clinical and demographic information, including prescription drug use, was gathered. The relationships between HADS scores and clinical factors were evaluated using Pearson’s correlation. **Results:** According to the HADS assessment, 20% of respondents had no anxiety symptoms, 17% had borderline symptoms, and 63% of patients reported definite anxiety symptoms. Of those with depression, 24% showed no symptoms, 14% were borderline, and 62% were certain. The average HADS-A and HADS-D scores were 2.34 and 2.43, respectively. L-DOPA alone was used to treat half of the patients, while combinations of Trivastal, Sifrol, anticholinergics, or antidepressants were given to the other half. There were no discernible correlations between HADS scores and clinical or demographic traits. **Conclusions:** The HADS is a useful instrument for assessing anxiety and depression in PD patients. Regardless of the method of treatment or stage of the disease, psychiatric symptoms are prevalent. For PD patients to benefit from early interventions and achieve an improved quality of life, routine screening is crucial.

## 1. Introduction

Parkinson’s disease is defined by motor symptoms, such as resting tremors, movement disorders or bradykinesia, and muscle rigidity, alongside non-motor symptoms, such as sleep and cognitive disorders. One of the main non-motor symptoms is the presence of anxiety disorders, which appears in 40% of Parkinson’s patients [1]. The prevalence of clinical disturbances ranges from 25 to 50% for anxiety [2,3,4] and 3 to 89% for depression [2,3,5]. Anxiety is one of the most common concerns for Parkinson’s patients; it impacts their quality of life [6,7,8] and can contribute to an increase in suicidal tendencies [9,10].

Different methods have been used to assess the presence or absence of anxiety. For example, Roy Byrne et al. [11] utilized a pre-established questionnaire based on the Diagnostic and Statistical Manual of Mental Disorders, Third Edition, Revised (DSM III R) criteria. They found that 16 out of 210 patients from the general population exhibited episodic anxiety, 8 patients met the criteria for panic anxiety, and among 47 patients, only 6 exhibited depression. The results showed that there is a different distribution of anxiety disorders, with higher rates of panic and anxiety compared with the general population.

The degradation of dopaminergic, serotonergic, and noradrenergic pathways, all of which are essential for mood regulation and motor control, may contribute to the high incidence of anxiety and depression in Parkinson’s disease. Emotional anguish can be worsened by psychological reactions to challenges brought on by the condition, such as social isolation, communication problems, and loss of autonomy. Therefore, it is conceivable that a combination of neurological and psychological factors makes this population more susceptible to anxiety and depression. These theories support the need for sensitive and targeted instruments to detect and screen for mental health issues in Parkinson’s disease patients.

## 2. Method

### 2.1. Samples

According to the UK Brain Bank criteria, 100 patients with Parkinson’s were included in this study, including 62 men and 38 women. Between March 2024 and May 2025, participants attended consecutive neurology consultations at the Mohammed VI University Medical Center and the Avicenne Military Hospital in Marrakech.

The inclusion criteria were as follows: age ≥ 18 years, Parkinson’s disease diagnosis, and the capacity to provide informed consent. To reduce the influence of recent surgery on mental symptoms, patients who had received deep brain stimulation (DBS) during the previous six months were not included. The Hoehn and Yahr scale was used to gauge the intensity of motor symptoms [12]. Written informed consent was provided by each participant, and this study received institutional ethics committee approval.

### 2.2. Procedure

An offer for participation was extended to eligible patients during their regular clinical appointment. They were requested to voluntarily fill out the questionnaire after being briefed on the goals and methods of the study during a special explanation session. Participation was completely voluntary and unpaid.

One known disadvantage of this study is that we did systematically record the number of eligible patients who declined to participate.

### 2.3. Anxiety Assessment Scales

Anxiety and depression symptoms were screened using the Hospital Anxiety and Depression Scale (HADS) [13]. A 4-point Likert scale (0–3) was used to rate each of the 14 items in the set. Two distinct scores, ranging from 0 to 21, were obtained from seven items that evaluate anxiety (subscale A) and seven items that evaluate depression (subscale D).

The scores are interpreted as follows:

0–7: Absence of symptoms;
8–10: Doubtful symptoms;
≥11: Certain symptomatology.

In neurological and medical populations, the HADS has shown strong validity and internal consistency.

### 2.4. Statistical Analyses

SPSS version 20.0 (IBM, Armonk, NY, USA) was utilized for the analysis of all the data. The sample was described using descriptive statistics, such as means, standard deviations, and frequencies.

The mean HADS scores of men and women were compared using Student’s *t*-tests for independent samples. The association between age, disease stage (Hoehn and Yahr score), and HADS scores was evaluated using Pearson’s correlation coefficients. 

The HADS thresholds were used to determine the prevalence rates of depression and anxiety. *p*-Values less than 0.05 were regarded as statistically significant.

## 3. Results

**Figure 1:**
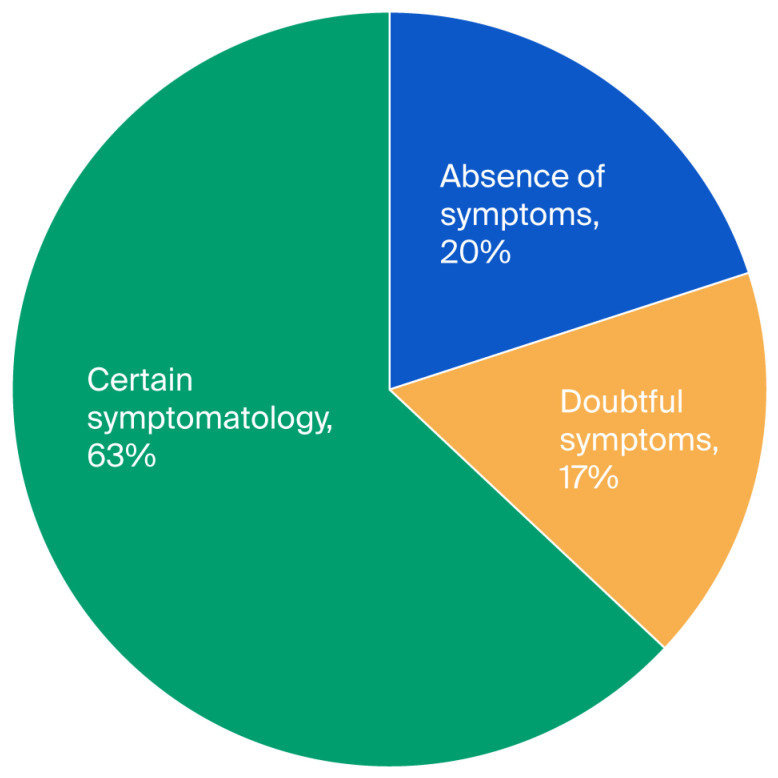
Distribution of anxiety among patients with Parkinson’s disease according to the three levels (*N*= 100).

**Figure 2:**
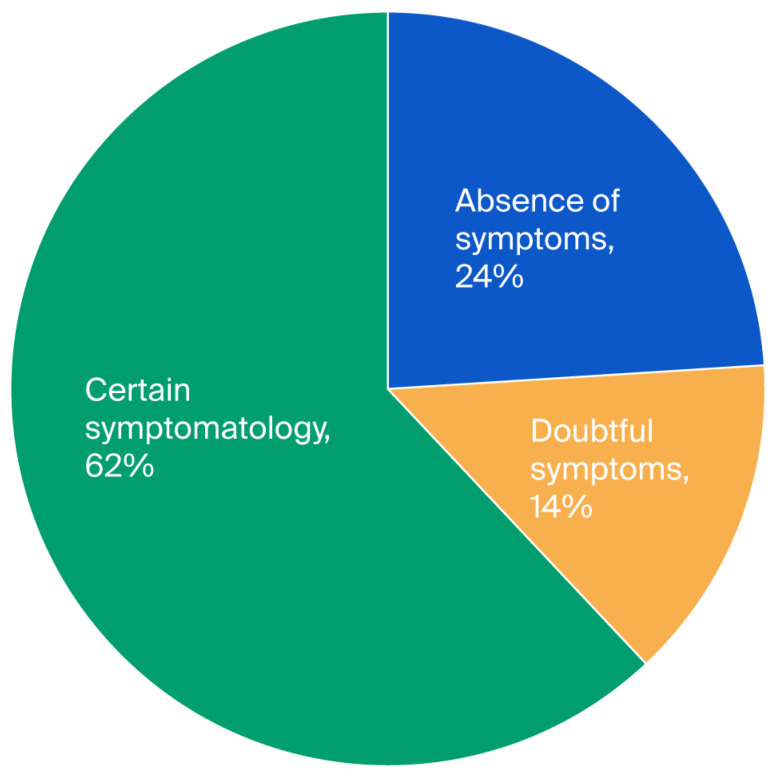
Distribution of depression among patients with Parkinson’s disease according to the three levels (*N*= 100).

This study consisted of 100 Parkinson’s patients who met the inclusion criteria, with 62 men (62%) and 38 women (38%), and the average age was 63.09 years. The average stage of the disease according to the Hoehn and Yahr scale (H & Y) was 2.76, and the average duration of the disease was 3.60 years. In our cohort, 9% of patients presented with unilateral disease, 40% presented with bilateral disease, 41% presented with bilateral disease and postural instability, and only 10% of patients were divided between two groups, one with a severe disability and the other in a wheelchair (see Table 1). There was no significant difference in relation to gender in terms of age (*p* = 0.72) and the disease stage (*p* = 0.65). To study anxiety, a score for each subscale of the Hospital Anxiety and Depression Scale (HADS) was applied. The three levels of anxiety observed were 20% for the first level (absence of symptoms), 17% for the second level (doubtful symptoms), and 63% for the third level (certain symptoms) (see Figure 1). In the case of depression, we found 24%, 14%, and 62% for the first, second, and third levels, respectively (see Figure 2); hence, the values obtained were close. Moreover, we found that the averages of anxiety and depression were 2.34 and 2.43, respectively (see Table 1).

In regard to medicine, among the 100 patients who were part of this study, half were treated with L-DOPA alone, and the other half were treated with a combination of L-DOPA and 50 mg of Trivastal. Furthermore, 20% of patients received prescriptions for L-DOPA at doses ranging from 0.25 mg to 2.1 mg in conjunction with Sifrol. Thirty percent of patients were receiving antidepressant therapy, while ten percent of the cohort utilized anticholinergic drugs. The fact that some patients were receiving multiple treatments simultaneously explains the overlapping percentages. This distribution emphasizes how difficult it is to treat Parkinson’s disease pharmacologically, especially when it comes to treating both motor symptoms and neuropsychiatric side effects like anxiety (see Table 2).

We did not find a significant difference in the average scores for the Hospital Anxiety and Depression Scale (HADS) between genders (for anxiety, t = −0.25 and *p* > 0.05; for depression, t = −1.64 and *p* > 0.05). Thus, for the relationships between the HADS, age, disease stage, and duration, the magnitude of the correlations ranged from 0.010 to 0.175 (*p* > 0.05).

We studied the elements that constitute the Hospital Anxiety and Depression Scale (HADS) to identify the specific components of anxiety and depression in our patient cohort. We calculated the percentage of patients who scored between 0 and 3 on the HADS. This type of analysis showed that feelings of worry, nervousness, and panic are among the most dominant anxiety states in patients. Thus, the inability to laugh easily, dissatisfaction with appearances, the sensation of being slowed down, and the absence of motivation are the main characteristics of depression (see Table 2). We used the Pearson test to study the correlation between anxiety, depression, and the disease stage and found that there is a correlation between depression and the disease stage.

Two different subscales that measure anxiety and depression independently make up the Hospital Anxiety and Depression Scale (HADS). Items 1, 3, 5, 7, 9, 11, and 13 make up the HADS-A (anxiety) subscale, and Items 2, 4, 6, 8, 10, 12, and 14 make up the HADS-D (depression) subscale. Each subscale offers a separate assessment of the associated emotional domain, enabling participants to differentiate between symptoms associated with depression and anxiety.

The item-by-item distribution of responses to the 14 HADS questions for each of the 100 study participants is shown in Table 3. A four-point Likert scale, with 0 signifying a lack of symptoms and 3 signifying the highest intensity of the symptom stated, was used to rate each item. Therefore, depending on the item’s content, higher scores indicate higher levels of anxiety or depression.

The table shows how many participants chose each of the following response options for each question: 0, 1, 2, or 3. As a result, each row represents a distinct HADS item, and the columns show the distribution of participant responses across the various answer categories.

Most participants chose scores of 0 or 1 for the majority of categories, indicating low to moderate symptom levels overall, according to the distribution of responses. Anxiety-related items, like “I feel tense or wound up” (Item 1) and “I’m worried” (Item 5), tended to have slightly higher answer rates in the 1–2 range, indicating that a subset of the population had mild to moderate anxiety. On the other hand, depressed statements like “I feel like I’m functioning slowly” (Item 8) and “I no longer care about my appearance” (Item 10) were more frequently evaluated as 0 or 1, suggesting that overall depressive symptoms were less severe.

## 4. Discussion

With average scores of 2.34 for anxiety and 2.43 for depression on the Hospital Anxiety and Depression Scale (HADS), our study showed that anxiety is very common among Parkinson’s patients. Although it is sometimes assumed that the intensity of motor symptoms plays a role in emotional distress, our results showed no significant correlation between HADS scores and clinical characteristics (disease stage) or demographic factors (age, sex, and length of disease). This implies that the development of motor symptoms in Parkinson’s disease may not be the only cause or the direct cause of anxiety and depression. However, clinical findings show that mental distress can result from a loss of mobility and autonomy, especially the inability to walk on one’s own. Social disengagement, anxiety about leaving the house, and feelings of discontent are typical and can feed a vicious cycle of psychological deterioration and loneliness. Patients’ mental health may be further impacted by these variables, which may interfere with daily routines and lower functional productivity.

Additionally, our findings corroborate earlier studies that suggested mood abnormalities in Parkinson’s disease are complex. As people gradually adjust to their condition, anxiety may gradually decrease, despite the fact that it may be significant immediately after diagnosis. This supports the notion that non-motor symptoms, such depression and anxiety, must be assessed and treated separately from the intensity of motor symptoms.

Although there is considerable heterogeneity, our results seem to be largely comparable with recent international studies regarding the frequency of anxiety and depression in our Moroccan group. While a significant percentage of patients in our study still displayed questionable or certain symptoms, indicating the presence of subclinical or developing mood disorders, 20% of patients reported no symptoms of anxiety, and 24% reported no symptoms of depression according to the HADS. The importance of these emotional disruptions in Parkinson’s disease is supported by recent research. The significance of screening for these non-motor symptoms is further supported by a 2022 meta-analysis that found that the prevalence of depression among Parkinson’s patients is approximately 38% worldwide [14]. In a similar vein, a cross-sectional survey carried out in Pakistan in 2022 discovered that 41.03% of patients had anxiety, and 33.3% had depression [15]. In a different 2021 study, 53.3% of PD patients had anxiety, which frequently co-occurs with depression and has a major negative influence on quality of life [16].

The cohort differences may be explained by assessment instruments, access to psychiatric care, and cultural views of mental health. Social stigma and a lack of adequate mental health infrastructure in Morocco may cause people to underreport emotional symptoms, which could result in lower recorded prevalence rates. These similarities emphasize the need for culturally appropriate mental health practices in Parkinson’s care as well as the usefulness of validated instruments like the HADS, which is sensitive to non-somatic symptoms. In our study, anxiety was primarily defined by concern, anxiousness, and panic, whereas depression was characterized by psychomotor slowness, a lack of desire, difficulty laughing, and dissatisfaction with appearance. Using the same questionnaire, Mondolo et al. [17] discovered similar anxiety traits, like agitation and difficulty relaxing, emphasizing restlessness as a key component of anxiety in Parkinson’s disease.

Additionally, they found that the Hamilton Anxiety Rating Scale (HAM-A) and the State–Trait Anxiety Inventory (STAI) do not adequately capture patients’ moods over the course of a week, although the Hospital Anxiety and Depression Scale–Anxiety subscale (HADS-A) does. Our results confirm that the HADS-A is a useful tool for assessing enduring emotional symptoms in Parkinson’s disease. Although they suggested additional measures, such as the Hamilton Depression Rating Scale (Ham-D), for diagnosing depression, Leentjens et al. [18] also showed that the HADS was sufficient for anxiety screening. In a sizable PD cohort, Marinus et al. [19] verified the reliability of the HADS. The Beck Anxiety Inventory (BAI) was less focused on cognitive anxiety symptoms than the Chinese Profile of Mood States (POMS) [20].

All things considered, these investigations demonstrate the advantages and limitations of several scales in PD, bolstering the HADS-A for assessing anxiety while pointing to the necessity for other instruments to thoroughly characterize depression.

The complex neurochemical mechanisms of anxiety in Parkinson’s disease are highlighted by the interactions of dopamine, norepinephrine, and serotonin. Dopaminergic and serotonergic combinations are frequently used to regulate mood and motor function, and frequent polypharmacy treatments addressed both motor and non-motor complaints in our population. Such regimens, however, may throw off the balance of neurotransmitters and raise the risk of interactions, which emphasizes the need for customized care and further investigation into how they affect anxiety in PD.

Pharmacological therapy for Parkinson’s disease (PD) must be carefully optimized, especially when treating neuropsychiatric symptoms like anxiety. The conventional treatment for motor symptoms, L-DOPA, was administered to all patients in this investigation. L-DOPA may also influence mood and anxiety through noradrenergic, serotonergic, and dopaminergic pathways [21]. Pramipexole (Sifrol) and other dopamine agonists can elevate moods and lessen depressive symptoms [22]; however, in susceptible patients, excessive dopaminergic stimulation may exacerbate anxiety [23].

Half of the patients in our group used L-DOPA and Trivastal together, which may have had indirect anxiolytic effects by improving cognitive function [24]. Ten percent of cases involved the prescription of anticholinergic drugs, which should be used with caution since they can exacerbate anxiety or disorientation [25]. In order to treat comorbid mood or anxiety disorders, 30% of patients were also taking antidepressants, primarily SSRIs. Although these substances are usually harmless, they should be monitored for interactions with dopaminergic medications [26]. The complicated nature of treating anxiety in PD is demonstrated by the frequent usage of several medicines.

Although polypharmacy raises the risk of interactions and probably reflects attempts to strike a balance between mood stabilization and motor control, it also highlights the necessity of customized treatment plans [27].

## 5. Limitations

It should be noted that this study has a number of limitations. First, judgments about causal relationships between the observed variables are limited by the study’s cross-sectional nature. Although correlations between anxiety, depression, and other variables can be found, it is impossible to determine how these relationships change over time. Future longitudinal research would be required to ascertain whether some variables may be regarded as predictors rather than correlations and to investigate how these psychological outcomes change over time.

Second, there are limitations to the findings’ generalizability. Due to its small size and recruitment from just two clinical sites, the study sample is not a true representation of the Moroccan community as a whole. When extrapolating the results to the national level, care should be used because sociocultural, regional, and healthcare access variations within Morocco may affect the prevalence and manifestation of anxiety and depressive symptoms.

Lastly, future studies should take into account longer-term designs, the incorporation of structured clinical interviews or mixed methods approaches, and larger and more varied samples from other geographical areas in order to move beyond these methodological limitations. Such research would increase the findings’ reliability and offer a more thorough comprehension of anxiety and depression in the Moroccan setting.

## 6. Conclusions

Parkinson’s disease patients frequently struggle with anxiety and depression, which has a major negative impact on their quality of life. In this study, we generated important regional insights by characterizing these symptoms in a Moroccan group. Pharmacological therapies are crucial for controlling motor symptoms, but they may have varying effects on neuropsychiatric symptoms, which highlights the need for tailored care and close monitoring. Early diagnosis and screening facilitate prompt action and increase patient–provider communication. Better outcomes overall may result from routine neuropsychiatric evaluations incorporated into Parkinson’s disease treatment. Additionally, our results highlight the necessity of culturally sensitive therapeutic practices. In order to improve care for this population, future research should concentrate on intervention trials to evaluate the effectiveness of specific treatments and longitudinal studies to track the development of symptoms.

## Figures and Tables

**Table 1:** Demographic and clinical features of PD patients (*N* = 100).

	Mean	Standard Deviation
Age (years)	63.09	8.50
Duration of Illness (years)	3.60	1.36
Stages of Illness According to (Hoehn and Yahr Scale)	2.76	0.98
The Hospital Anxiety and Depression Scale (HADS) Scores for Anxiety	2.34	0.78
Depression	2.43	0.80

**Table 2:** Parkinson’s disease patients’ medication distribution (*N* = 100).

Medication	Number of Patients	Percentage (%)
L-DOPA	50	50%
L-DOPA + Trivastal (50 mg)	50	50%
L-DOPA + Sifrol (0.25 mg–2.1 mg)	20	20%
Anticholinergics	10	10%
Antidepressants	30	30%

**Table 3:** Item-by-Item analysis of the HADS.

The Items	0	1	2	3
1. I feel tense or ‘wound up’.	27	50	9	14
2. I take pleasure in things than before.	12	37	33	18
3. I have a feeling of fear as if something horrible is going to happen to me.	1	39	9	33
4. I laugh easily and see the bright side of things.	9	40	32	19
5. I’m worried.	37	42	7	14
6. I am in a good mood.	23	46	12	19
7. I can sit quietly and do nothing and feel casual.	10	36	25	29
8. I feel like I’m functioning slowly.	57	22	11	10
9. I feel sensations of fear, and I have a knot in my stomach.	42	30	9	19
10. I no longer care about my appearance.	17	57	13	13
11. I have ants in my pants and can’t sit still.	37	42	12	9
12. I am looking forward to the idea of doing certain things.	10	50	22	18
13. I experience sudden sensations of panic.	15	29	19	37
14. I can take pleasure in a good book or a good radio show or television.	26	25	24	25

## Data Availability

The datasets generated during this study are available from the corresponding author upon reasonable request.
